# Could axillary clearance be avoided in clinically node-negative breast cancer patients with positive nodes diagnosed by ultrasound guided biopsy in the post-ACOSOG Z0011 era?

**DOI:** 10.1371/journal.pone.0210437

**Published:** 2019-01-10

**Authors:** Miao Liu, Yang Yang, Fei Xie, Jiagia Guo, Siyuan Wang, Houpu Yang, Shu Wang

**Affiliations:** Breast Center, Peking University People's Hospital, Beijing, China; University of Campinas, BRAZIL

## Abstract

**Background and objectives:**

The role of ultrasound (US) guided biopsy in selecting patients for an upfront lymph node dissection (ALND) has been controversial following the publication of the American College of Surgeons (ACOSOG) Z0011 data. The purpose of this study was to investigate if patients with positive axillary lymph nodes (LNs) proven by US guided biopsy should be recommended for ALND and to analyze the utility of preoperative US and US guided biopsy in planning axillary surgery to avoid both unnecessary ALND and unnecessary SLNB.

**Methods:**

Patients with a positive preoperative LN biopsy were identified and evaluated to determine their suitability for inclusion according to the criteria proposed in the Z0011 data. The correlation of the number of suspicious nodes found using US with the number of positive nodes on ALND was studied.

**Results:**

A total of 261 breast cancer patients who had a positive preoperative LN needle biopsy were identified, among them, 79 patients with cT1–2N0 breast cancer and ALND were enrolled in the study. Thirty-one patients (39.2%) had ≤2 positive nodes identified in pathology and 10 patients (12.7%) met all of the Z0011 criteria and might have been spared ALND. A significantly greater proportion of women with ≥3 positive nodes during ALND had >1 abnormal LN identified using US compared to women with ≤2 positive LNs found using ALND (66.7% vs. 6.5%, *p*<0.0001).

**Conclusion:**

US with needle biopsy is valuable to patients with multiple suspicious nodes found using US while SLND without US guided needle biopsy is suggested if only one abnormal LN is detected on US in the post-Z0011 era.

## Introduction

Axillary LN status is the most important prognostic factor in breast cancer. SLNB is currently the standard of care for staging clinically negative axilla in breast cancer patients [1–3].

Preoperative axillary US has been widely used for evaluating the clinical nodal status in breast cancer. If the patient’s axillary evaluation is negative, an SLNB is performed for further staging. If a suspicious LN is detected with US, this node will then be sampled and if pathology shows a metastasis, the SLNB is omitted and an ALND is indicated [4–8]. Thus, implementation of US guided biopsy can reduce the number of patients who need to undergo unnecessary SLNB, which is beneficial for the patient and has been shown to reduce health care costs [9].

However, the role of US guided biopsy in selecting patients for an upfront ALND has been questionable after publication of the American College of Surgeons (ACOSOG) Z0011 data. This trial demonstrated that in clinically node -negative women undergoing breast-conserving therapy (BCT) who were found to have metastases in 1 or 2 SLNs, SLNB alone resulted in rates of local control, disease-free survival (DFS), and overall survival (OS) equivalent to those seen after ALND but with significantly lower morbidity [10–11]. Although some studies have confirmed that patients with axillary LN metastasis identified by US guided biopsy had more positive LNs compared with patients with SLND-identified nodal metastases [12–13], US guided biopsy may not appropriately identify clinically node negative women who require ALND [14]. Among the patients with positive nodes diagnosed by US guided biopsy, there maybe a sub-group of patients who fulfil the inclusion criteria for the Z0011 study, and they are likely over-treated if managed with upfront ALND.

Therefore, the first aim of this study was to investigate if patients with positive LNs identified by US guided biopsy should be recommended for ALND. Second, we sought to identify the method for predicting ALND properly with US and US guided biopsy in patients with clinically negative but positive nodes diagnosed by US guided biopsy to avoid both unnecessary ALND and unnecessary SLNB.

## Materials and methods

### Study patients

Breast cancer patients with a positive preoperative axillary LN needle biopsy were retrospectively identified from July 2007 through December 2017 at the Breast Center of Peking University People’s Hospital (PKUPH).

Patients who had palpable axillary disease, had a pathologic tumor size >5 cm, presented with an axillary recurrence, did not undergo ALND or had received neoadjuvant chemotherapy were excluded. A waiver was issued by PKUPH’s review board, exempting the requirement to obtain ethics approval before submission of the manuscript. Written consent was obtained from all the patients.

### Pre-operative US and needle biopsy

In accordance with PKUPH guidelines, all breast cancer patients underwent axillary US as a routine workup before operation by a group of breast radiologists. The US appearance of the axillary LNs was classified as benign or suspicious. US criteria for suspicious lymphadenopathy included cortical thickness >3 mm, eccentric cortical thickening, round or lobulated shape of the LN, loss of fatty hilum, and unclear margins. The number of suspicious LNs seen on US was categorized as 1or >1 node according to the US report. Patients whose records described multiple suspicious LNs without defining an absolute number, using terminology such as ‘several’ or ‘many’, were characterized as having >1 suspicious node.

US guided biopsy was performed by fine needle aspiration (FNA) in most cases using a 24-gauge needle and occasionally by 16-gauge core needle biopsy (CNB) at the discretion of the radiologist. In cases in which multiple suspicious nodes were present, only the most suspicious node was sampled. Cytological samples of the LN were stained with a Papanicolaou staining and with a Giemsa staining for cytological analysis. Histological analysis was performed by haematoxylin and eosin (H&E) staining and immunohistochemistry.

### Axillary surgery

When US guided biopsy results were positive (including malignant cells or highly suspicious malignant cells on pathology), an ALND was performed at primary surgery. When US guided biopsy results were negative, a standard SLNB was performed.

### Application of ACOSOG Z0011 criteria

All patients enrolled in this study were evaluated to determine their suitability for inclusion as proposed by the Z0011 study criteria, including T1 or T2; clinically node-negative invasive breast cancer; one or two positive SLNs by routine H&E staining or frozen section; and treatment with BCT, whole breast irradiation (WBI), and adjuvant systemic therapy (chemotherapy and/or endocrine therapy). Patients were ineligible if they had 3 or more positive nodes on ALND. Patients who could not undergo standard adjuvant treatment after surgery were ineligible. Furthermore, any patients who underwent a mastectomy were also deemed ineligible. Once all the criteria were met, the number of patients who could have been spared ALND was calculated.

### Data and statistical analysis

Patient and tumor characteristics including age, tumor size, tumor histology, nuclear grade, presence of lymphovascular invasion, estrogen and progesterone receptor status, HER2/neu amplification, type of surgery, total number of resected LNs, and the total number of positive LNs were collected from a prospective institutional database.

The total number of positive axillary LNs was categorized as minimal nodal involvement (≤2 positive nodes) or extensive nodal involvement (≥3 positive lymph nodes), as proposed by the Z0011 trial.

The percentage of patients who should have avoided ALND was calculated according to the Z0011 criteria. The false negative percentages and negative predictive values (NPVs) for axillary nodal staging according to Z0011 criteria with US were calculated. Chi-squared tests and Fisher’s exact tests were used for categorical variables to analyze the relativity of the number of suspicious nodes on US with the number of positive nodes on ALND. SPSS22.0 software was used for statistical analysis. A *P* value of <0.05 was considered significant.

## Results

**Fig 1:**
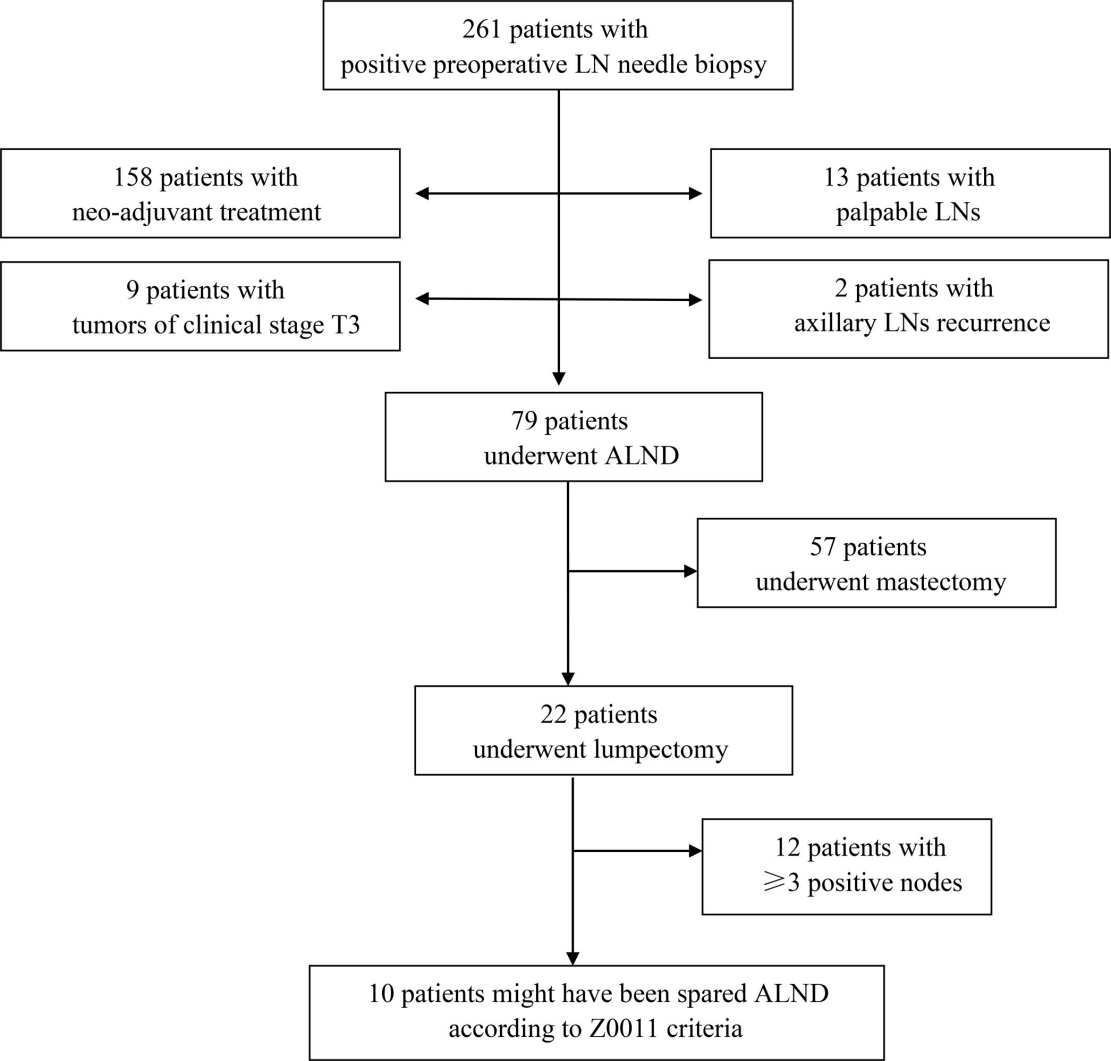
Flow chart of the study procedures. (LN-lymph node; ALND-lymph node dissection).

Between July 2007 and December 2017, a total of 261 breast cancer patients who had a positive preoperative LN needle biopsy were identified. Ten patients (3.8%) had core needle biopsy, and the others had fine needle biopsy. Of these women, 158 patients who had neoadjuvant treatment, 13 patients who had palpable LNs, 9 patients who had clinical stage T3 tumors (tumor size >5cm) and 2 patients who had axillary LNs recurrence were excluded, leaving 79 patients in the study population (Fig 1). All the 79 patients were diagnosed with LN metastasis by fine needle biopsy, before they underwent breast surgery.

The median patient age for the cohort was 57 years (range 26–88 years), and the median tumor size was 2 cm (range: 0.4–5.0 cm). The majority of patients had an invasive ductal carcinoma (62/79; 78.5%), 4 patients (5.0%) had invasive lobular histology, and 13 (16.5%) had mixed pathology. Thirty-six patients (45.6%) were grade II, 33 patients (41.8%) were grade Ⅲ, and lymphovascular invasion was present in 45 patients (57.0%). A total of 69.6% tumors were estrogen receptor positive (55/79), while 21 (26.6%) were Her-2 receptor positive, and 8 patients (10.1%) were triple negative.

The median number of nodes removed at axillary clearance was 20 (range: 10–47). The median number of positive nodes excised during ALND was 4 (range: 1–27). Thirty-one patients (39.2%) had ≤2 positive nodes, and among them, 23 patients had 1 positive node and 8 patients had 2 positive nodes. Of the 79 patients, 22 patients (27.8%) underwent lumpectomy and 57 patients (72.2%) underwent mastectomy. Of the 22 patients who had undergone lumpectomy, 10 patients (45.5%) had ≤2 positive nodes. Therefore, of the 79 patients enrolled in this study, there were 10 patients (12.7%) who would have met the criteria for inclusion in the Z0011 study (Fig 1).

**Fig 2:**
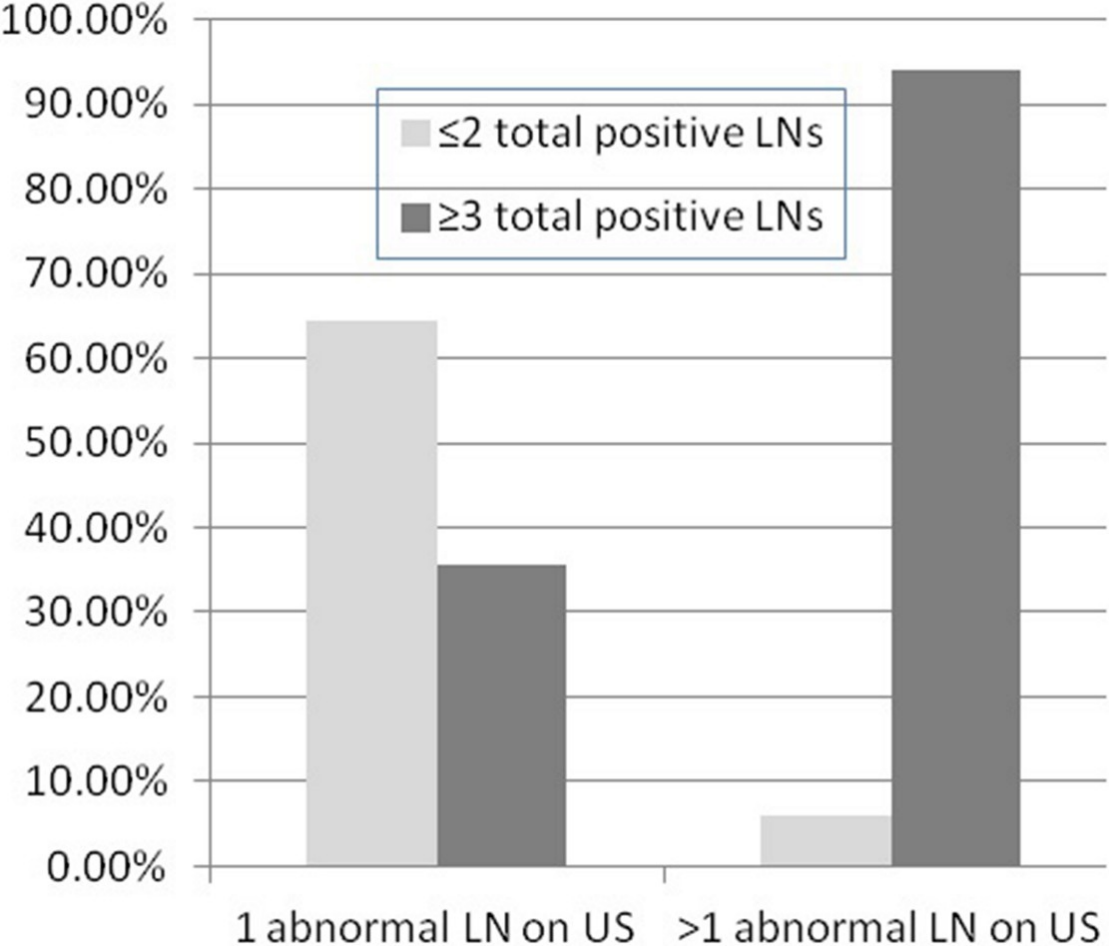
Positive lymph node (LN) involvement for women with 1 or >1 abnormal axillary LN identified by ultrasound (US).

We further examined preoperative axillary US imaging by whether solitary node or multiple abnormal axillary LNs were identified. Visualizing one versus multiple suspicious LNs on US predicted minimal nodal involvement (≤2 pos tive nodes) or extensive nodal involvement (≥3 positive lymph nodes) on final pathology as follows: there were 45 patients with only one abnormal LN on US, and among them, 29 patients (64.4%) had ≤2 positive LNs, and the pathologic N stage was N1 in 32 patients (71.1%) and N2 in 13 patients (28.9%) (Fig 2). Thus, extensive nodal involvement (≥3 positive LNs) was present in 16 of 45 axillae with solitary abnormal LN on US, resulting in a false negative percentage of 35.6% (16/45) and an NPV of 64.4% (29/45) to exclude advanced nodal disease (≥3 positive LNs) in this subgroup of patients with only one abnormal LN predicted by axillary ultrasound.

In contrast, for US guided biopsy-positive patients with multiple abnormal LNs on imaging, 2 (5.9%) of 34 patients had ≤2 positive LNs and the pathologic N stage was N1 in 9 patients (26.5%), N2 in 18 patients (52.9%) and N3 in 7 patients (20.6%) (Fig 2).

A significantly greater proportion of women with ≥3 total positive nodes during ALND had >1 abnormal LN identified on preoperative axillary US compared to women with only 1 or 2 total positive LNs (66.7% vs. 6.5%, *p* <0.0001) at final pathology (Table 1).

**Table 1:** Relativity of suspicious LNs on US with positive LNs burden on pathology.

suspicious LNs on US	≤2 positive LNs on pathology (n = 31)	≥3positive LNs on pathology (n = 48)	*p*
1	29(93.5%)	16(33.3%)	<0.0001
>1	2(6.5%)	32(66.7%)	

LNs, lymph nodes; US, ultrasound.

## Discussion

Current guidelines from the National Comprehensive Cancer Network (NCCN) recommend SLNB for patients with clinically negative LNs and fine or core needle biopsy for patients with clinically positive LNs [15]. In the NCCN guideline, clinically positive axilla was defined by physical examination alone. However, preoperative axillary ultrasound is widely used for assessing the nodal status in breast cancer patients with clinically negative nodes. If the patient’s axillary evaluation on US is negative, an SLNB is performed for further staging. If a suspicious lymph node is detected with US, this node will then be sampled, and if pathology shows a metastasis, the SLNB is omitted and an upfront ALND is indicated [14,16–17].

Axillary US with needle biopsy has a positive predictive value (PPV) for the detection of nodal metastases approaching 100%. The application of US and needle biopsy for evaluating axillary node status has been due to the accumulation of evidence regarding this approach, along with its efficiency, feasibility, relative simplicity and the modest cost of this staging strategy. In Hieken et al.’s series [18], preoperative US with needle biopsy for suspicious axillary LNs proved valuable for operative treatment planning by permitting ALND without SLNB in 28.6% of node-positive patients and in 8.6% of newly diagnosed patients with invasive breast cancer who were treated with primary surgical therapy overall. Leenders et al.’s study found that the preoperative identification rate of axillary metastases by axillary US and needle biopsy was 24.7%, with a consequent reduction in SLNB of 9.2% [12]. A meta-analysis of 31 studies on US-guided biopsies of axillary LNs in breast cancer patients by Houssami showed that the median proportion of women triaged directly to ALND was 19.8% [14].

In our center, preoperative US assessment of the axilla with needle biopsy of suspicious nodes has become routine practice in the past ten years. The benefits of this workup, which include omitting unnecessary SLNB for node-positive breast cancer patients, reducing time spent in waiting for frozen pathology during the operation, reducing the cost of frozen section examination, and avoiding a second operation for patients, have been reported in our previous study [19].

While the management of breast cancer is changing rapidly, with the goal of optimal oncologic safety and minimizing surgical morbidity for the patients. The results from the ACOSOG Z0011 trial caused a paradigm shift in axillary management of invasive breast cancer. According to the Z0011 trial, breast cancer patients who had 1or 2 positive SLNs, and were treated with breast conservational surgery, adjuvant systemic therapy and whole breast irradiation could be spared an ALND. Based on these findings, the current guidelines from the American Society of Clinical Oncology (ASCO) and the NCCN recommend considering no further surgery for patients who meet ACOSOG Z0011 eligibility criteria [15]. Many centers in the United States, Europe and Australia have applied the Z0011 criteria to their populations [20–23] since the publication of Z0011. Our center investigated the feasibility of applying the Z0011 criteria to Chinese patients [24] and has been the first to incorporate Z0011 results into clinical practice in China since 2014.

However, the role of US with needle biopsy has been challenged after application of Z0011 data to the treatment of patients. The questions mainly focus on whether ALND is recommended for every metastasis detected by US guided needle biopsy and how to identify patients for an upfront ALND by ultrasound with needle biopsy.

Morrow et al. [25] found that 66 (47%) of 141 patients with positive LNs diagnosed by US and needle biopsy before operation had only 1 to 2 total positive LNs and 26 patients (18.4%) could be safely managed with SLNB alone if treated according to ACOSOG Z0011 criteria. Boland et al. [26] reported that 134 (38.6%) of 347 patients identified with positive US/FNA had less than three positive nodes and that 27 patients (7.8%) satisfied the criteria proposed by the Z0011 group and potentially could have been spared ALND. Caudle et al. [27] showed that 52% (99/190) of patients whose US with needle biopsy showed positive disease had ≤2 positive lymph nodes identified in their ALND specimens, and a report from the Mayo Clinic indicated that 48.4% of patients who had axillary nodal metastases identified by US had ≤2 positive nodes on surgical pathology [18]. In this study, among 79 patients with positive LNs diagnosed by US guided needle biopsy, 31 patients (39.2%) had ≤2 positive nodes, and a total of 10 patients (12.7%) were eligible for inclusion of the Z0011 criteria and did not require ALND. It is encouraging to note that, of 22 patients who had undergone lumpectomy, 10 patients (45.5%) fully matched the Z0011 criteria and might have been spared ALND if they had undergone an SLNB procedure (Fig 1).

Our data are consistent with others and indicate that not all of the patients who have metastasis in LNs detected by US guided needle biopsy should undergo ALND, because there is a sub-group of patients among them who fulfil the Z0011 criteria, meaning that SLNB is safe and sufficient for them.

However, some researchers [18,27,28] have stated that the ACOSOG Z0011 trial criteria are only applicable to patients with a positive axilla found by SLNB, not to patients with disease identified by US. Patients with positive nodes on US and biopsy had significant differences in surgical pathologic findings compared with those having positive nodes on SLNB, including more additional positive lymph nodes and larger metastases [29–30].

Intuitively, it seems logical that patients with more axillary tumor burden are more easily discovered or identified by radiologists than those with less tumor load. But data from former studies [25–27] and the current study all indicate that, among the patients who had positive LNs on preoperative US with biopsy, the number of patients who could have been avoided unnecessary ALND according to Z0011 is not negligible. In our opinion, ALND should not be recommended for every metastasis detected by US guided needle biopsy in the post-ACOSOG Z0011 era.

Though preoperative diagnosis of axillary node metastases using US with biopsy has high specificity and PPV, it is impossible to stage axilla as accurately as SLNB [18,27]. Hence, since the ACOSOG Z0011 trial results were published, there has been discussion about limiting US guided LN biopsy to patients who have suspicious-looking LNs [12,31]. However, US with biopsy, especially FNA, is quick to perform, well tolerated and has limited adverse effects. Two meta-analyses [14,32] have indicated that preoperative axillary US with selective biopsy will correctly identify approximately 50% of breast cancer patients who have axillary node metastases, and it is noteworthy that preoperative axillary US and biopsy as a staging strategy to triage women with node metastases to ALND could remove unnecessary SLNB. The key issue is how to differentiate between minimal nodal disease (1–2 metastatic nodes) and a greater burden of nodal disease using US with biopsy before operation.

We tried to analyze the relativity of the number of suspicious nodes on US with the number of positive nodes on final pathology. As a result, we found that 64.4% of patients with only one abnormal LN on US had ≤2 positive LNs, 71.1% were at stage N1, and 28.9% were with stage N2. For US guided biopsy- positive patients with multiple abnormal LNs on imaging, 5.9% of them had ≤2 positive LNs and the pathologic N stage was 26.5% with N1, 52.9% with N2 and 20.6% with N3. A significantly greater proportion of women with ≥3 total positive nodes during ALND had >1 abnormal LN identified on preoperative axillary US compared to women with only 1 to 2 total positive LNs (65.3% vs. 6.5%, *p*<0.0001) at final pathology. Morrow et al. [25] reported that women with >1 abnormal LN by US were more likely to have≥3 positive total LNs than women with only 1 abnormal LN identified by axillary imaging (68% vs. 43%, *p* = 0.003). Hieken et al. [18] also compared final nodal pathology for women with 1 vs. >1 abnormal LN on preoperative axillary imaging and found that for US with needle biopsy- positive patients, visualizing multiple versus one abnormal LN on US predicted 3 or more positive LNs on final pathology (70.0% vs 33.3%, *p* = 0.0008). These data indicated that >1 abnormal LN on US is a predictor of higher nodal disease burden.

**Fig 3:**
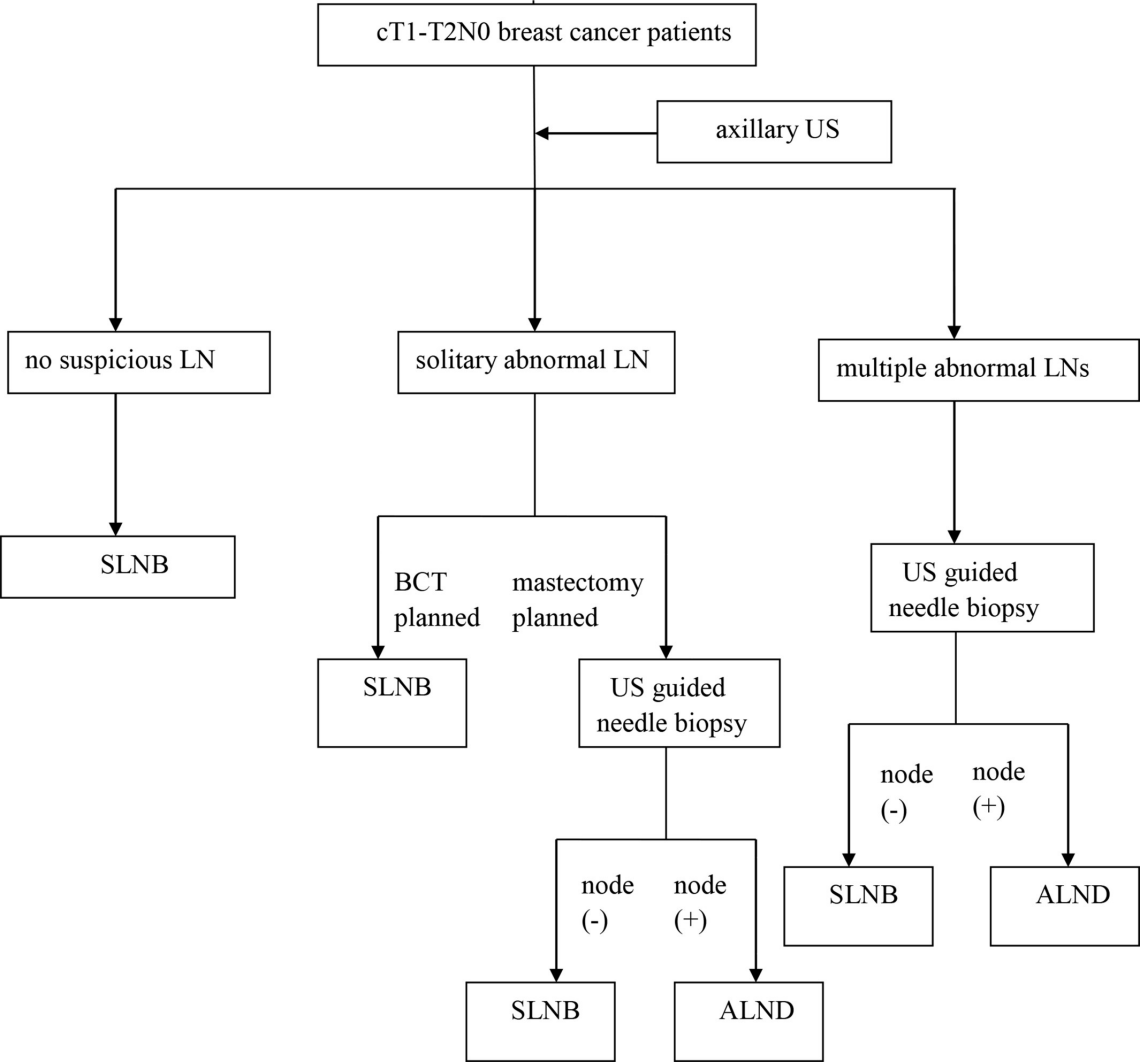
An algorithm for assessment of cT1-T2N0 breast cancer patients with preoperative US.

Therefore, we suggest assessing cT1-T2N0 breast cancer patients with preoperative US to make the axillary surgical plan as follows: 1. If no suspicious node is found on US, SLNB is recommended; 2. If solitary abnormal LN is observed on US and BCT is planned, SLNB is recommended without the need for needle biopsy, because there is a strong probability that quite a portion of this group of patients will fulfil the Z0011 criteria and ALND can be avoided. If solitary abnormal LN is observed on US and a mastectomy is planned for the patient, US with biopsy is proposed, and a positive result indicates the need for an ALND; 3. If multiple abnormal LNs are detected with US, the most suspicious node should be sampled, disregarding the breast surgical mode. If pathology shows a metastasis, the SLNB should be omitted and an upfront ALND is recommended (Fig 3).

Gathering information regarding the status of axillary nodes prior to surgical intervention can assist in making a treatment plan. Preoperative examination using imaging with imaging-directed needle biopsy to detect and confirm nodal metastases can triage surgical management of the axilla, avoiding unnecessary operation. Evaluating an imaging modality should take into account its accuracy, and more importantly its clinical utility. US with biopsy has emerged as the most practical technique for assessment of the axilla in breast cancer patients with clinically negative LN, mainly due to its efficiency, feasibility, relative simplicity and modest cost. Continued refinements of imaging techniques, such as elastography [33] and microbubbles [34], may improve performance by accurately identifying the SLN prior to surgery.

## Conclusion

Our study has some limitations, including its retrospective nature and small sample size. However, our center has been playing a leading role in adopting Z0011 criteria into clinical practice in China, and to our knowledge, this study is the first to discuss the utility of US with needle biopsy for axillary operative planning after the publication of Z0011 in our country. Our data support that not all patients with a positive axillary node needle biopsy should be recommended for an upfront ALND. US with needle biopsy is valuable to patients with multiple suspicious nodes on US, and SLND without the need for US guided needle biopsy is suggested if only one abnormal LN is detected on US in the post- Z0011era.
